# Characterization of 7-Methylguanosine Identified Biochemical Recurrence and Tumor Immune Microenvironment in Prostate Cancer

**DOI:** 10.3389/fonc.2022.900203

**Published:** 2022-05-23

**Authors:** Sheng Xin, Yuxuan Deng, Jiaquan Mao, Tao Wang, Jihong Liu, Shaogang Wang, Xiaodong Song, Wen Song, Xiaming Liu

**Affiliations:** ^1^ Department of Urology, Tongji Hospital, Tongji Medical College, Huazhong University of Science & Technology, Wuhan, China; ^2^ Institute of Urology, Tongji Hospital, Tongji Medical College, Huazhong University of Science & Technology, Wuhan, China

**Keywords:** prostate cancer, 7-methylguanosine, lncRNAs, biochemical recurrence, tumor immune microenvironment, prognostic model

## Abstract

Prostate cancer (PCa) has a high incidence rate, mortality rate, and biochemical recurrence (BCR) rate. 7-Methylguanosine (m7G), as one of the RNA modifications, has been considered to be actively involved in cancer-related translation disorders in recent years. Therefore, we first used The Cancer Genome Atlas (TCGA) database to identify prognosis and m7G-related long non-coding RNAs (lncRNAs). Then we randomly divided the samples into the training set and test set and then constructed and verified the m7G lnRNA prognostic model (m7Gscore) by the least absolute shrinkage and selection operator (LASSO) regression analysis. The m7Gscore has been proved to be an independent marker of BCR-free survival in patients with PCa. Furthermore, the m7Gscore was significantly correlated with the tumor immune microenvironment (TIME) and somatic mutation of PCa patients and had the potential to be an indicator for the selection of drug treatment. We also clustered TCGA cohort into three m7G-related patterns (C1, C2, and C3). The Kaplan–Meier survival analysis revealed that C1 had the best BCR-free survival and C3 had the worst. The TIME was also significantly distinct among the three m7G-related patterns. According to the TIME characteristics of the patterns, we defined C1, C2, and C3 as immune-desert phenotype, immune-inflamed phenotype, and immune-excluded phenotype, respectively.

## Introduction

Prostate cancer (PCa) has the highest morbidity and mortality in the genitourinary system malignant tumor, with approximately 248,530 new cases and 34,130 deaths in the United States in 2021 (1). Radical prostatectomy (RP) and radiotherapy are considered the standard treatment for localized PCa (2, 3). However, about 20%–40% of prostate patients will have a biochemical recurrence (BCR) in traditional treatment and may eventually develop into castration-resistant PCa (CRPC) (4). At present, the main basis of PCa risk assessment includes clinical stage, pathological grade, and prostate-specific antigen (PSA) level, but the clinical evidence suggests that these are not enough to accurately evaluate the prognosis of PCa patients (5, 6). Therefore, screening molecular markers with prognostic value and constructing a more accurate and specific risk model are of great significance to improve the prognosis and diagnosis of PCa patients (7, 8). Moreover, due to individual differences and side effects, the expected efficacy of drug therapies, such as androgen deprivation therapy (ADT), chemotherapy, and immunotherapy, are not consistent for each PCa patient. Therefore, an effective treatment prediction model will have significant benefits for the prognosis of patients with PCa.

Long non-coding RNAs (lncRNAs) are RNA transcripts that are longer than 200 nucleotides and do not code for proteins (9). They have a stronger tissue-specific expression pattern than mRNA (10). LncRNAs have the functions of mediating signaling pathways, translation programs, and post-transcriptional gene expression control in cancers (11–13). They can also be used as markers for tumor diagnosis and prognosis (14). RNA modification is crucial in the regulation of post-transcriptional (15). 7-Methylguanosine (m7G) modification widely exists in eubacteria, eukaryotes, and some archaea (16). The mRNAs of most eukaryotic cells need to be translated by relying on the m7G cap at its 5′ end (17, 18). Eukaryotic translation initiation factor 4E (eIF4E) is the target of the most vital signaling pathways, PI3K/Akt/mTOR and RAS/MAPK in PCa, which mediate the tumor progression. The role of eIF4E in many mRNA translation initiations needs to be mediated by binding with the m7G cap (19). It is reported that ribavirin can compete with the m7G cap to bind eIF4E, reduce the translation ability of the eIF4E complex, and reduce tumor proteins such as Mcl-1, to play an antitumor role (20). Therefore, targeting the m7G cap may be of great significance to prevent PCa from progressing to CRPC.

To develop the BCR predictive value of m7G-related lncRNAs in PCa, 408 samples were selected from The Cancer Genome Atlas (TCGA) database and randomly divided into the training set and test set. A prognostic model (m7Gscore), which can accurately and stably predict the BCR-free survival in PCa patients, was constructed according to the training set. It has a significant correlation with multiple clinicopathological features and has been proved to be an independent prognostic index. We also established a nomogram model including the m7Gscore and several clinicopathological features to more accurately predict BCR-free survival. Both the m7Gscore and the nomogram model were validated in the test set and the entire TCGA cohort. The m7Gscore was shown to have a significant predictive ability for tumor immune microenvironment (TIME), somatic mutation, and drug treatment effect in patients with PCa. Additionally, TCGA cohort was clustered according to the expression of hub lncRNAs constituting the m7Gscore. Then we analyzed the differences in BCR status and TIME among distinct m7G-related patterns.

## Method

### Data Collection and Processing

RNA-sequencing (RNA-seq) data and clinical information of 501 PCa patients were retrieved from TCGA database (**Supplementary Table 1**) (21). A total of 93 patients without complete BCR information were removed. There were 391 PCa patients with single-nucleotide variation (SNV) data. The expression of the gene symbol with multiple Ensembl IDs was referred to as the average value. The format of RNA-seq information was fragments per kilobase of transcript per million mapped reads (FPKM). RNA-seq information was performed in log transformation. A total of 408 samples were divided into the training set and test set according to 1:1 by using the “caret” package in R (**Supplementary Table 2**).

A total of 29 m7G-related mRNAs were collected from three m7G-related gene sets in the Molecular Signatures Database (MSigDB) (http://www.gsea-msigdb.org/gsea/msigdb/index.jsp), including GOMF_M7G_5_PPPN_DIPHOSPHATASE_ACTIVITY, GOMF_RNA_7_METHYLGUANOSINE_CAP_BINDING, GOMF_RNA_CAP_BINDING, and the reported review (**Supplementary Table 3**) (16). Then 388 m7G-related lncRNAs were screened according to the criteria of |Cor| > 0.4 and p-value <.001. The co-expression network of lncRNAs and mRNAs was constructed by the “igraph” package in R.

### Development and Assessment of the 7-Methylguanosine Model

The m7G-related lncRNAs expression and BCR-free survival information of the training set were matched, and the univariate Cox regression model was constructed to extract the prognosis-related lncRNAs (p < 0.05). Then the least absolute shrinkage and selection operator (LASSO) regression analysis further selected variables and determined coefficients with one SE above the minimum criteria, which were performed *via* the “glmnet” R package (22). The formula for risk score was


m7Gscore=∑(exp Genei×coefficient Genei)

According to the m7Gscore and the optimal cutoff by the “surv_cutpoint” R function, samples were differentiated into the high or low risk. The Kaplan–Meier survival analysis and time-dependent receiver operating characteristic (ROC) curve (“survival” and “timeROC” packages) were performed to evaluate the predictive ability of the m7Gscore. The area under the curve (AUC) was used to quantify the ROC curve. To validate the model, the test set and TCGA cohort were analyzed with the same cutoff value and analysis methods.

### Establishment and Evaluation of the Nomogram Model

Based on the training set, a nomogram model was constructed by incorporating the m7Gscore and clinicopathological factors to estimate the 1-, 3-, and 5-year BCR-free survival. The univariate and multivariate Cox regression analyses were enrolled to calculate the coefficient. The nomogram for TCGA cohort was constructed with the “rms” R package and used calibration analysis to assess the accuracy. The concordance index (C-index), the Kaplan–Meier curves, and ROC curves evaluated the effectiveness.

### Unsupervised Consensus Clustering

To further explore the biological characteristics of m7G in PCa patients, the “ConsensusClusterPlus” package in R was used to cluster the patients with the expression of m7Gscore-related lncRNAs (23). The number of iterations was 50, and the resampling rate was 80%. Finally, the clustering results were selected based on the cumulative distribution function (CDF) curve and relative change in the area. The principal component analysis (PCA) was performed with the “prcomp” function in R.

### Functional Enrichment Analysis

The “clusterProfiler,” “enrichplot,” and “DOSE” R packages were used to perform gene set enrichment analysis (GSEA). GSEA was used to research the biological characteristics of the m7Gscore-defined groups. It transformed the expression matrix of isolated genes into specific gene sets as the expression of biological process characteristics (24). After the samples were divided into two groups, the GSEA algorithm calculated the fold change (FC) of each gene expression between them and then ranked genes according to the FC. If a gene belonged to a specific pathway, its running enrichment score (ES) = FC/sum FC. If not, running ES = −1/(total number of genes − the number of genes in this pathway) and then accumulated continuously in order. When the maximum is reached, it is the ES of this pathway. The specific gene sets for GSEA were downloaded from the MSigDB (http://www.gsea-msigdb.org/gsea/msigdb/index.jsp).

### Analysis of the Tumor Immune Microenvironment

ESTIMATE and single-sample GSEA (ssGSEA) were performed to evaluate the correlations between the m7Gscore and TIME. The ssGSEA algorithm investigated the enrichment score of different tumor-infiltrating immune cell (TIIC) subsets and immune functions. The ESTIMATE algorithm analyzed the immune and stromal cell content to evaluate the TIME of each sample. Additionally, the TIIC infiltration results in PCa were comprehensively calculated with various immune analysis algorithms in TCGA cohort, including XCELL, TIMER, QUANTISEQ, MCPCOUNTER, EPIC, CIBERSORT-ABS, and CIBERSORT (25–30).

### Chemotherapy and Endocrine Therapy Drug Response

The Genomics of Drug Sensitivity in Cancer (GDSC) was used to evaluate the effectiveness of PCa patients to 8 chemotherapeutic and endocrine therapy drugs with the “pRRophetic” R package (31). The ridge regression was used to estimate the 50% inhibitory concentration (IC50) and to evaluate the accuracy by 10× cross-validation (32).

### Statistical Analysis

R software (version 4.1.2) was used to perform all statistical analyses in this study. Fisher’s exact test and chi-square test were used to determine the significance of distributed differences in clinicopathological stages. Since there were discrete values in the m7Gscore, a log transfer for the m7Gscore was performed to reduce the influence of outliers and better display the distribution (**Supplementary Table 4**). The Wilcoxon rank-sum test and Kruskal–Wallis test were used to compare continuous variables between two or more groups. Pearson’s correlation analysis was employed to calculate the correlation coefficient between variables. In the correlation analysis of the m7Gscore, a log transfer for the m7Gscore was performed. The heatmaps in our study were constructed *via* the “pheatmap” R package. The values represented by each cell in the heatmap were centralized and standardized with Z-score. The “ggalluvial” R package was used to build Sankey diagrams for visualizing the attributes between variables. After the SNV data of TCGA-KIRC cohort were downloaded, the tumor mutation burden (TMB) of each sample was calculated *via* Perl scripts (version 5.34.1). TMB was defined as the sum of all somatic mutations in the coding area. Mutation spectrums were displayed by constructing the waterfall diagram *via* Perl and the “maftools” R package. Significant statistical significance was defined as p-value <0.05 (*p < 0.05; **p < 0.01; ***p < 0.001; ns = no significance).

## Result

**Figure 1:**
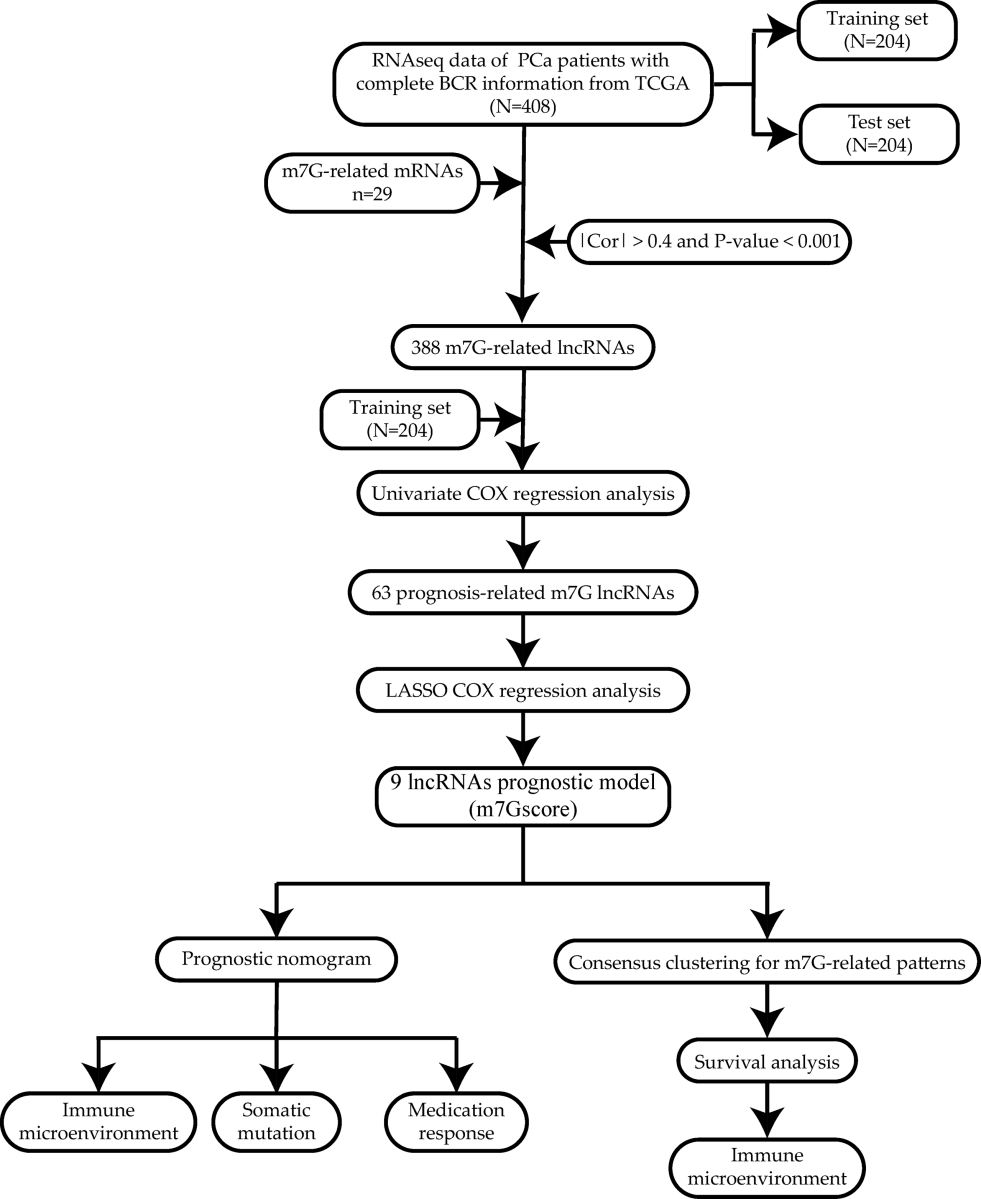
Workflow of the study.

The workflow is shown in **Figure 1**.

### Identification of 7-Methylguanosine-Related Long Non-Coding RNAs

**Figure 2:**
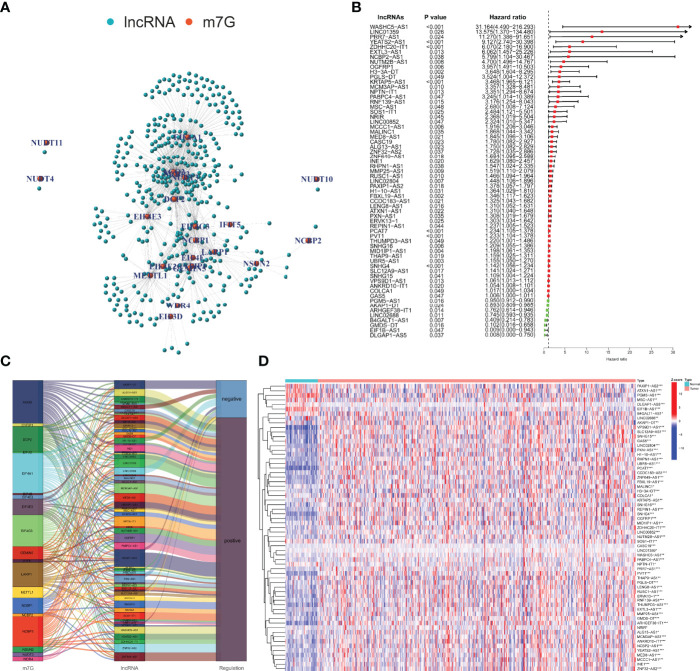
Identification and evaluation of the m7G-related lncRNAs. **(A)** Co-expression network of m7G-related mRNAs and lncRNAs. **(B)** Selection of prognosis-related lncRNAs with univariate Cox regression analysis. **(C)** The correlation between m7G-related mRNAs and lncRNAs with the Sankey diagram. **(D)** Expression difference analysis of the m7G-related lncRNAs. m7G, 7-methylguanosine; lncRNAs, long non-coding RNAs. *p < 0.05; **p < 0.01; ***p < 0.001.

According to the MSigDB and previous literature, we sorted out 29 m7G-related mRNAs. With the use of the RNA-seq data of PCa samples in TCGA, 388 m7G-related lncRNAs were screened based on |Cor| > 0.4 and p-value <0.001 (**Figure 2A**). **Figure 2B** shows 63 m7G-related lncRNAs included in the univariate Cox regression model (p < 0.05). We used the Sankey diagram to visualize the correspondence and regulation of m7G-related mRNAs and lncRNAs in **Figure 2C**. The Wilcoxon rank-sum test demonstrated the m7G-related lncRNAs with significant differences between PCa and normal tissues (**Figure 2D**).

### Construction of the 7-Methylguanosine Model

We first randomly divided TCGA cohort into the training set and test set. Through the statistical analysis, we confirmed that there was no significant difference in the distribution of clinicopathological features (**Table 1**).

**Table 1:** Difference analysis of clinicopathological characteristics between the training set and test set.

Characteristic	Training	Test	p-Value	Method
n	204	204		
T stage, n (%)			0.246	Fisher’s test
T2	82 (20.3%)	68 (16.9%)		
T3	114 (28.3%)	132 (32.8%)		
T4	4 (1%)	3 (0.7%)		
N stage, n (%)			0.495	Chisq test
N0	142 (39.9%)	148 (41.6%)		
N1	36 (10.1%)	30 (8.4%)		
Gleason score, n (%)			0.876	Chisq test
6	16 (3.9%)	21 (5.1%)		
7	104 (25.5%)	98 (24%)		
8	26 (6.4%)	25 (6.1%)		
9	57 (14%)	58 (14.2%)		
10	1 (0.2%)	2 (0.5%)		
PSA	0.1 (0.03, 0.1)	0.1 (0.03, 0.15)	0.556	Wilcoxon
Age	61 (56, 66)	62 (56, 66)	0.712	Wilcoxon

PSA, prostate-specific antigen.

**Figure 3:**
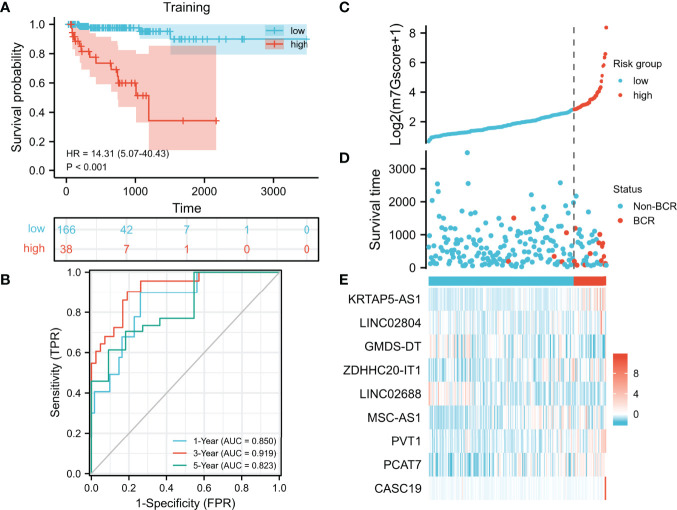
Development and evaluation of the m7G model. **(A, B)** Evaluation of the m7G model with Kaplan–Meier analysis **(A)** and time-dependent ROC curves **(B). (C–E)** Distribution of m7Gscore **(C)**, BCR status **(D)**, and model consisting of m7G-related lncRNAs **(E)**. m7G, 7-methylguanosine; ROC, receiver operating characteristic; BCR, biochemical recurrence.

In the training set, we used the LASSO regression analysis to further select hub m7G-related lncRNAs from the univariate Cox regression model (**Supplementary Figure 1**). Eventually, a total of 9 lncRNAs were included in the m7G model (m7Gscore). m7Gscore = (1.03977 × KRTAP5-AS1 expression) + (0.35534 × LINC02804 expression) − (1.13057 × GMDS-DT expression) + (1.32000 × ZDHHC20-IT1 expression) − (0.12685 × LINC02688 expression) + (1.61489 × MSC-AS1 expression) + (0.20102 × PVT1 expression) + (0.12749 × PCAT7 expression) + (0.26826 × CASC19 expression). The optimal cutoff value (cut point = 6.12870) partitioned the training set into the high- and low-risk groups. The high-risk patients tended to have significantly worse BCR-free survival (**Figure 3A**, hazard ratio [HR] = 14.31 (5.07–40.43), p < 0.001). The AUC of the ROC curves was 0.850, 0.919, and 0.823, which suggested that the m7Gscore performed well (**Figure 3B**). Consistently, the distributions of the m7Gscore and BCR status showed distinct differences between the m7Gscore-defined groups (**Figures 3C, D**). The expression of KRTAP5-AS1, LINC02804, ZDHHC20-IT1, MSC-AS1, PVT1, PCAT7, and CASC19 was positively correlated with the m7Gscore, while GMDS-DT and LINC02688 expressions were negatively correlated with the m7Gscore (**Figure 3E**).

### Validation of the 7-Methylguanosine Model

**Figure 4:**
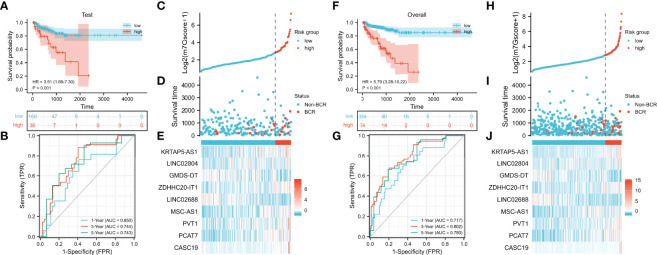
Validation of the m7G model. **(A, B)** Evaluation of the m7G model with Kaplan–Meier analysis **(A)** and time-dependent ROC curves **(B)** in the test set. **(C–E)** Distribution of m7Gscore **(C)**, BCR status **(D)**, and model consisting of m7G-related lncRNAs **(E)** in the test set. **(F, G)** Evaluation of the m7G model with Kaplan–Meier analysis **(F)** and time-dependent ROC curves **(G)** in TCGA cohort. **(H–J)** Distribution of m7Gscore **(H)**, BCR status **(I)**, and model consisting of m7G-related lncRNAs **(J)** in TCGA cohort. m7G, 7-methylguanosine; ROC, receiver operating characteristic; BCR, biochemical recurrence; TCGA, The Cancer Genome Atlas.

We verified the prognostic value of the m7Gscore in the test set and TCGA cohort. The m7Gscore formula and cut point were the same as the training set. The BCR-free survival of the low-risk patients was also significantly longer than that of the high-risk patients (**Figure 4A**, test set: HR = 3.51 (1.69–7.30), p = 0.001; **Figure 4F**, TCGA cohort: HR = 5.79 (3.28–10.22)). Moreover, the ideal validation was provided in the ROC curves of the test set with AUCs of 0.650, 0.744, and 0.743 (**Figure 4B**). The AUCs of TCGA cohort were 0.717, 0.802, and 0.780 (**Figure 4G**). The distribution heatmaps also showed similar results in the test set and TCGA cohort (**Figures 4C–E, H–J**).

### Clinical Correlation of the 7-Methylguanosine Model

**Figure 5:**
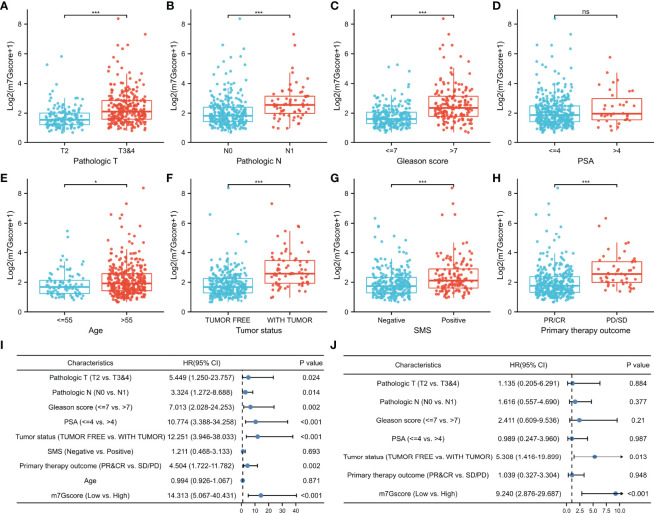
Association between m7Gscore and clinicopathological factors. **(A–H)** The distribution of m7Gscores according to pathological T stage **(A)**, N stage **(B)**, Gleason score **(C)**, PSA (**D**), age **(E)**, tumor status **(F)**, SMS **(G)**, and primary therapy outcome **(H). (I, J)** Verification of the characteristics of m7Gscore to be an independent BCR-free prognostic index with the univariate **(I)** and multivariate **(J)** Cox regression analyses. PSA, prostate-specific antigen; SMS, surgical margin status; BCR, biochemical recurrence. *p < 0.05; ***p < 0.001. ns, no significance.

Due to the difference in BCR-free survival, we compared the m7Gscore among different clinicopathological factors to investigate the clinical relevance of the m7G model in TCGA cohort. **Figures 5A–H** show that advanced pathologic TN stages, elevated Gleason score, aging, positive tumor status when a new tumor event occurred after treatment, positive surgical margin status (SMS), and bad primary therapy outcome were connected with a significantly higher m7Gscore.

In the univariate Cox regression model, it was found that TN stages, Gleason score, PSA, tumor status, primary therapy outcome, and m7Gscore were risk factors for the BCR-free survival of PCa patients (**Figure 5I**). After the multivariate Cox regression model was constructed, the m7Gscore was confirmed as the independent BCR-free prognostic parameter (**Figure 5J**).

### Development and Evaluation of the Nomogram Model

**Figure 6:**
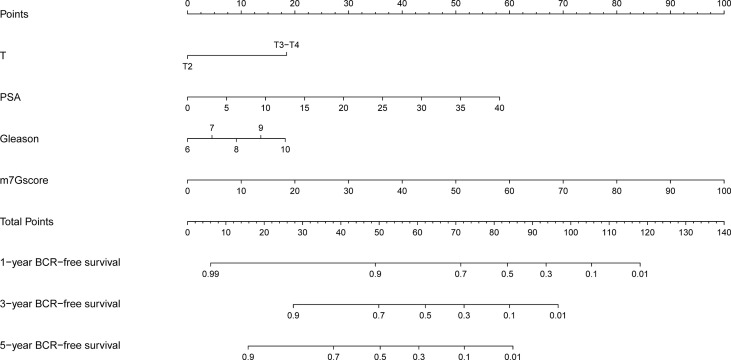
Development of the nomogram model.

Based on the clinicopathological characteristics and BCR information of PCa samples in the training set, we constructed a nomogram model by using univariate and multivariate Cox regression analyses. The patients with unavailable clinicopathological information were excluded. The nomogram was an establishment combining the m7Gscore and clinicopathological features to provide a novel and effective BCR-free survival prediction tool for the clinic (**Figure 6**).

**Figure 7:**
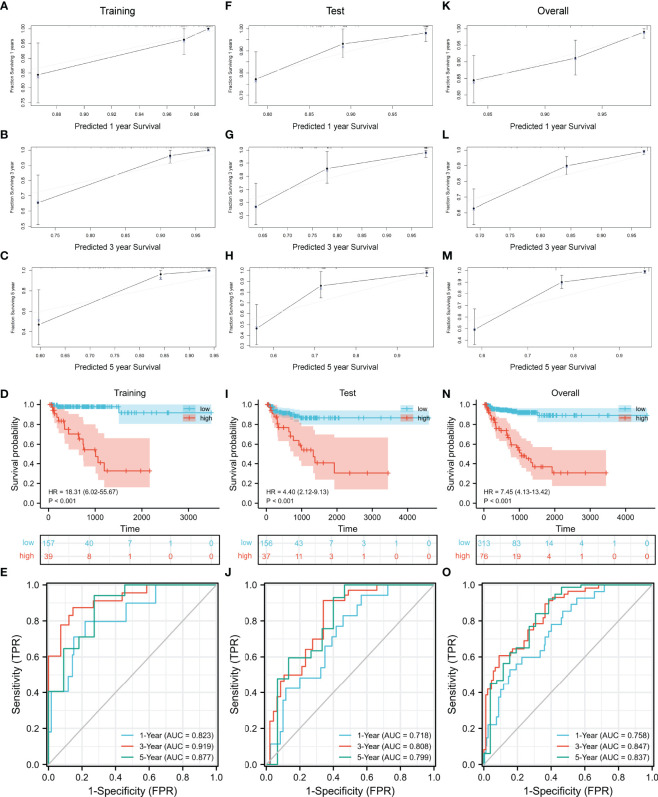
Evaluation and validation of the nomogram model. **(A–C)** Evaluation of the nomogram model with calibration curves in 1, 3, and 5 years. Evaluation of the nomogram model with Kaplan–Meier analysis **(D)** and ROC curves **(E)**. Validation of the nomogram model with calibration curves in the test set **(F–H)** and overall TCGA cohort **(K–M)**. Validation of the nomogram model with Kaplan–Meier analysis **(I)** and ROC curves **(J)** in the test set. Validation of the nomogram model with Kaplan–Meier analysis **(N)** and ROC curves **(O)** in the overall TCGA cohort. ROC, receiver operating characteristic; TCGA, The Cancer Genome Atlas.

Then we evaluated the accuracy and effectiveness of the nomogram model in the training set and verified the stability of the model by using the verification set and TCGA cohort. The calibration curves for 1-, 3-, and 5 years of survival showed good performance (**Figures 7A–C**), indicating accurate prognostic results. The C-index was 0.85874 (95% CI: 0.76911–0. 94836, p = 4.326E−15). The training set, test set, and overall set were split into the low and high groups according to the optimal cutoff value (cut point = 0.60809) based on the nomogram model. The high-risk patients in the training set had a significantly worse BCR-free survival (**Figure 7D**, HR = 18.31 (6.02–55.67), p < 0.001). The AUCs of time-dependent ROC curves were 0.823, 0.919, and 0.877 (**Figure 7E**).

As the result of the validation of the model, the calibration curves still showed a good fit (**Figures 7F–H, K–M**). The BCR-free survival of high-risk patients in both the test set and TCGA cohort was also significantly worse than that of low-risk patients (**Figure 7I**, test set: HR = 4.40 (2.12–9.13), p < 0.001; **Figure 7N**, TCGA cohort: HR = 7.45 (4.13–13.42), p < 0.001). The AUCs of the test set were 0.718, 0.808, and 0.799 (**Figure 7J**). In TCGA cohort, the AUCs were 0.758, 0.847, and 0.837 (**Figure 7O**).

### Co-Expression Relevance and Gene Set Enrichment Analysis

**Figure 8:**
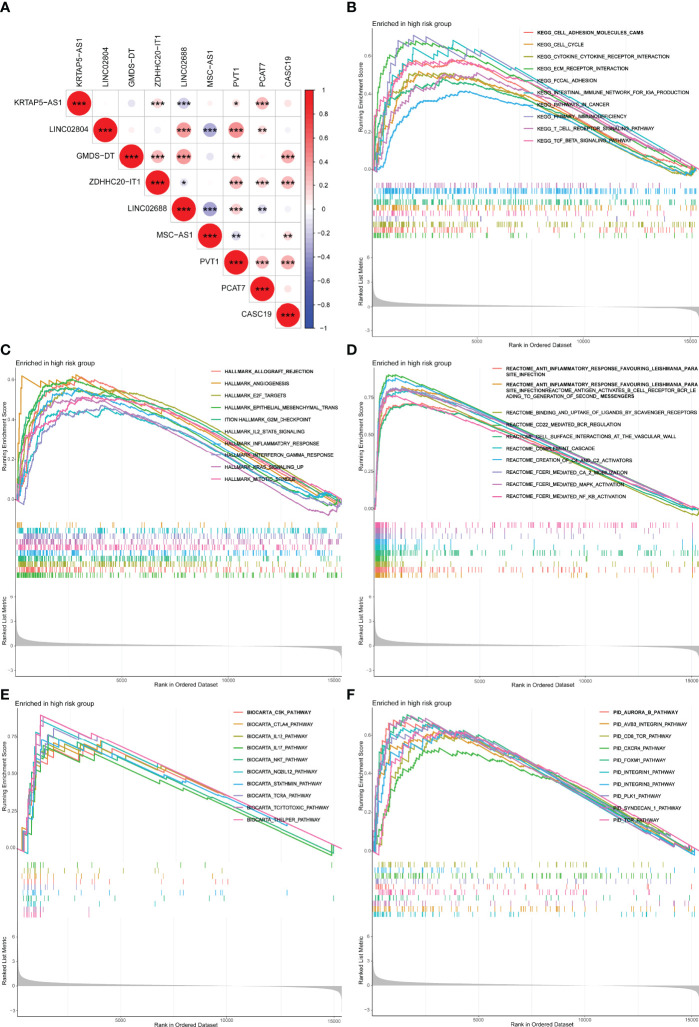
Co-expression network and functional enrichment analysis of m7Gscore-defined groups. **(A)** Co-expressed correlation among the hub m7G-related lncRNAs in PCa. **(B–F)** GSEA results of KEGG **(B)**, Hallmark **(C)**, Reactome **(D)**, BioCarta **(E)**, and PID **(F)**. lncRNAs, long non-coding RNAs; PCa, prostate cancer; GSEA, gene set enrichment analysis. *p < 0.05; **p < 0.01; ***p < 0.001.

The co-expressed correlation analysis of 9 m7G-related lncRNAs in PCa indicated that PVT1 was the center node with eight significant correlations (**Figure 8A**). PVT1 is the activator of MYC, an important oncogene in PCa. PVT1 was demonstrated to promote PCa growth by inhibiting apoptosis (33). Furthermore, we performed the GSEA with five distinct gene sets to research the underlying mechanisms of the m7G (**Figures 8B–F**). The results showed that the high m7Gscore phenotypes were significantly associated with several immune activation functions, including T-cell receptor signaling pathway, and cytokine–cytokine receptor interaction.

### Tumor Immune Microenvironment of the m7Gscore-Defined Groups

**Figure 9:**
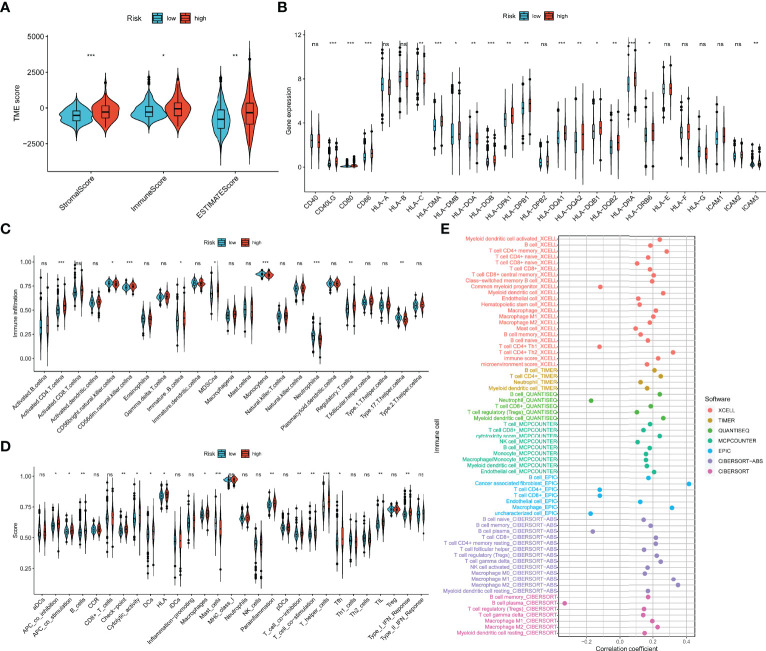
Association between the TIME and m7G model. **(A)** The ESTIMATE of tumor microenvironment in the two m7Gscore-defined groups. **(B)** MHC, costimulatory, and adhesion molecule expression in the m7Gscore-defined groups. **(C, D)** Enrichment indices of TIICs **(C)** and immune functions **(D)** in the m7Gscore-defined groups with ssGSEA. **(E)** Evaluation of the correlation between m7Gscore and the infiltration level of TIICs with comprehensive algorithms. TIME, tumor immune microenvironment; m7G, 7-methylguanosine; MHC, major histocompatibility complex; TIICs, tumor-infiltrating immune cells; ssGSEA, single-sample gene set enrichment analysis. *p < 0.05; **p < 0.01; ***p < 0.001. ns, no significance.

As shown in **Figure 9A**, the ESTIMATE algorithm revealed that immune, stromal, and ESTIMATE scores were distinct between m7Gscore-defined groups and elevated in the high-risk group. Most MHC and costimulatory molecules were also upregulated with the m7Gscore elevated (**Figure 9B**). **Figure 9C** reveals that the infiltration level of CD4+ T cells, CD56dim natural killer (NK) cells, immature B cells, myeloid-derived suppressor cells (MDSCs), and regulatory T cells (Treg) was higher in the high-risk patients, while immature CD56dim NK cells, monocytes, neutrophils, and type 17 T helper (Th17) cells were less infiltrated. The enriched scores in most immune functions of the high-risk tumor microenvironment (TME) were also higher (**Figure 9D**). Furthermore, we comprehensively revealed the correlation between log2(m7Gscore + 1) and the infiltration level of TIICs with multiple algorithms in **Figure 9E** and showed part of the correlation scatter diagram in **Supplementary Figure 2**. Collectively, the m7Gscore was more likely positively correlated with the fraction of B cells, memory CD4+ T cells, CD8+ T cells, macrophages, and myeloid dendritic cells (DCs) in TME.

### Association Between the 7-Methylguanosine Model and Somatic Mutation

**Figure 10:**
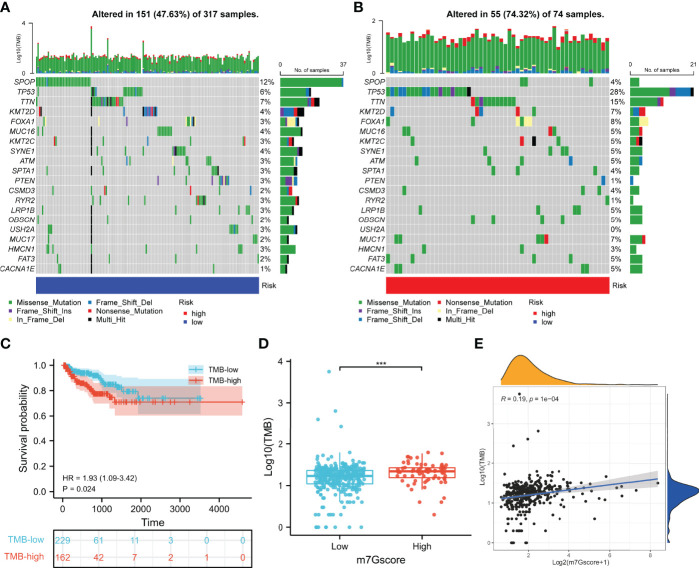
Somatic mutation of the m7Gscore-defined phenotypes. **(A, B)** Mutational spectrums consisted of the 20 most frequent mutation genes in the m7Gscore-defined groups. **(C)** Correlation between TMB and BCR-free survival of PCa patients. **(D, E)** Relevance between m7Gscore and TMB with box plot **(D)** and correlation analysis **(E)**. TMB, tumor mutation burden; BCR, biochemical recurrence; PCa, prostate cancer. ***p < 0.001.

To investigate the role of m7G in the somatic mutation, we calculated the mutational spectrum and TMB *via* SNV information of 391 patients in TCGA cohort (**Supplementary Table 5**). Due to the existence of discrete values, we performed a log10 transfer for TMB analysis. The waterfall diagram of the low- and high-risk groups showed the 20 most frequently mutated genes, among which SPOP and TP53 had the highest rate (**Figures 10A, B**). TCGA cohort was clustered into the TMB-low and TMB-high groups according to the optimal cutoff value (cut point = 19) calculated by the “surv_cutpoint” R function, and higher TMB indicated poorer BCR-free survival of PCa patients (**Figures 10C**). Consistently, the m7Gscore was positively correlated with TMB (**Figures 10D, E**).

### Drug Sensitivity of the m7Gscore-Defined Groups

**Figure 11:**
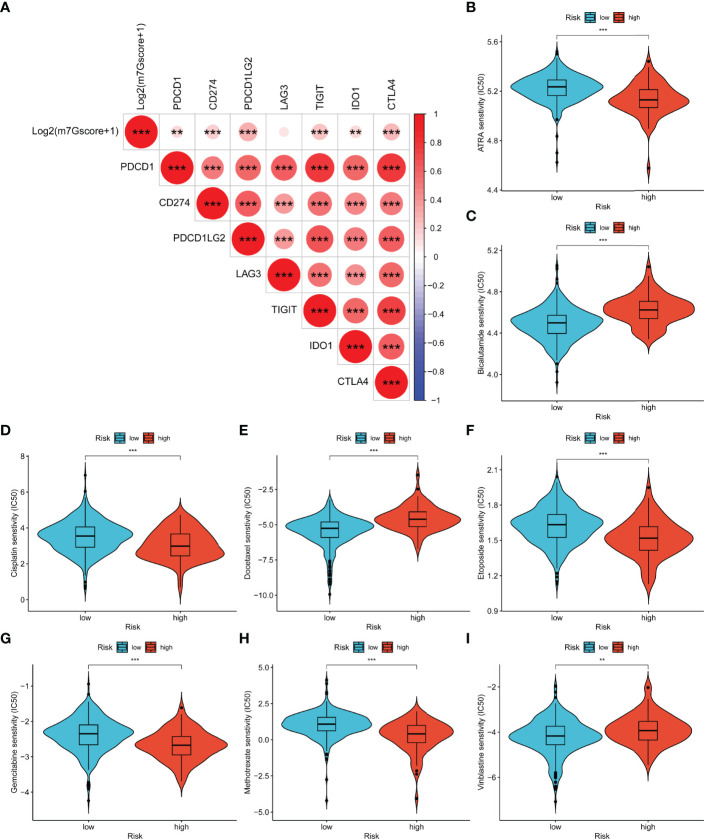
Relevance between m7Gscore and drug sensitivity. **(A)** The prediction of immunotherapy efficacy with the correlation between m7Gscore and immune checkpoint expression. **(B–I)** The IC50 of m7Gscore-defined subgroups to drugs, including ATRA **(B)**, bicalutamide **(C)**, cisplatin **(D)**, docetaxel **(E)**, etoposide **(F)**, gemcitabine **(G)**, methotrexate **(H)**, and vinblastine **(I)**. **p < 0.01; ***p < 0.001.

As shown in **Figure 11A**, log2(m7Gscore + 1) had significantly positive relevance with six immune checkpoints (PDCD1, CD274, PDCD1LG2, TIGIT, IDO1, and CTLA4). Consequently, we speculated that high-risk PCa patients tend to respond effectively to immunotherapy. Moreover, we evaluated the response of different subgroups to chemotherapy and endocrine drugs by IC50 values (**Figures 11B–I**). It was found that the IC50 value of bicalutamide, docetaxel, and vinblastine was significantly higher in the high-risk group. ATRA, cisplatin, etoposide, gemcitabine, and methotrexate were more suitable for low-risk patients.

### Identification of 7-Methylguanosine-Related Patterns by Consensus Clustering

**Figure 12:**
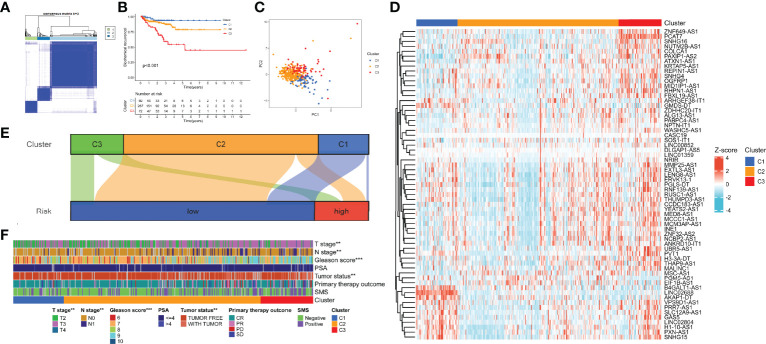
Identification of m7G-related patterns by consensus matrix factorization. **(A)** Consensus matrix. **(B)** BCR-free survival in m7G phenotypes with Kaplan–Meier curves. **(C)** The expression characteristics of m7G-related lncRNAs of m7G phenotypes with PCA diagram. **(D)** Expression of the m7G-related lncRNAs in m7G-related patterns. **(E)** Distribution of samples in m7G-related patterns in the high- and low-risk groups with Sankey diagram. **(F)** Correlations between m7G-related patterns and clinicopathological parameters. m7G, 7-methylguanosine; BCR, biochemical recurrence; lncRNAs, long non-coding RNAs; PCA, principal component analysis. **p < 0.01; ***p < 0.001.

According to the expression of the 9 hub lncRNAs, we categorized TCGA cohort into distinct patterns with consensus clustering. k = 3 was selected as the best clustering size (**Figure 12A**). C1, C2, and C3 consisted of 69, 267, and 72 patients, respectively. **Figure 12B** shows that C1 had the best BCR-free survival and C3 had the worst (p < 0.001). The result of PCA revealed a substantial difference in m7G transcription levels among m7G patterns (**Figure 12C**). The heatmap showed the expression profile of m7G-related lncRNAs in the patterns (**Figure 12D**). In the Sankey diagram, we found that most samples of C2 and C3 belonged to the low-risk group, while about half of C1 were high-risk (**Figure 12E**). Eventually, we calculated the clinical relevance of m7G-related patterns, and the composition of pathologic TN stages, Gleason score, and tumor status was significantly distinct (**Figure 12F**). Moreover, the advanced stages or events tended to concentrate on C3.

### Tumor Immune Microenvironment of 7-Methylguanosine-Related Patterns

**Figure 13:**
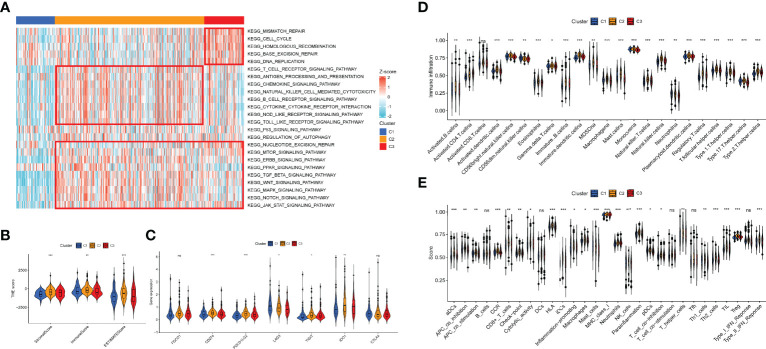
Association between the TIME and m7G-related patterns. **(A)** The GSEA result in DNA damage repair, immune activation, and carcinogenic biological functions. **(B)** The ESTIMATE result of the three m7G phenotypes. **(C)** The expression of immune checkpoints in the m7G patterns. **(D, E)** Enrichment indices of TIICs **(D)** and immune functions **(E)** in the m7G patterns. TIME, tumor immune microenvironment; m7G, 7-methylguanosine; GSEA, gene set enrichment analysis; TIICs, tumor-infiltrating immune cells. *p < 0.05; **p < 0.01; ***p < 0.001, ns, no significance.

To discover the effect of m7G on biological function regulation in PCa, we performed GSEA and found that C2 exhibited an elevated concentration in immune activation and carcinogenic functions, while C3 had highly expressed DNA damage repair and carcinogenic functions (**Figure 13A**). C1 showed lower enrichment levels in DNA damage repair, immune activation, and carcinogenic functions. Then we evaluated the constituent of TME to clarify the TIME difference among the three patterns (**Figure 13B**). As a result, C2 possessed higher stromal, immune, and ESTIMATE scores, which indicated lower tumor purity. Moreover, some immune checkpoints (CD274, PDCD1LG2, TIGIT, and IDO1) were upregulated in C2, while LAG3 was the most highly expressed in C1(**Figure 13C**). Then, the ssGSEA results indicated that the infiltration level of all TIICs was distinct among the patterns and C2 had the highest level (**Figure 13D**). Similarly, the expression of most immune functions was elevated in C2 (**Figure 13E**). **Supplementary Figure 3** shows the immune response heatmap obtained by the enrichment analysis algorithm based on TIMER, CIBERSORT, CIBERSORT-ABS, QUANTISEQ, MCPCOINTER, XCELL, and EPIC.

## Discussion

PCa is an inert tumor, but it has about 25% recurrence and metastasis rates, resulting in a high conversion rate of CRPC, high drug resistance, and low survival rate (34). Consequently, accurate and stable biomarkers are very important for identifying high-risk PCa and improving survival and treatment strategies. m7G tRNA has been shown to control the translation intensity to induce tumorigenesis and metastasis in a variety of cancers, including PCa (35–37). Therefore, the molecular mechanism of PCa progression based on m7G-related lncRNAs is worthy of further research (38). High-throughput sequencing and bioinformatics analysis have been more and more widely used in clinics to find potential genomic markers for diagnosis and prognosis (39). The research of tumor risk models can reduce the detection cost and improve the effectiveness of prediction. The systematic study of m7G-related lncRNAs will not only help to find their potential value in PCa but also contribute to determining specific and effective diagnostic and prognostic biomarkers.

In our study, 408 PCa samples with complete BCR information collected from TCGA database were included. To evaluate and quantify the m7G-related patterns of PCa patients and accurately predict the BCR-free survival, we randomly selected 204 samples from TCGA-PRAD dataset as the training set to construct a prognostic model (m7Gscore) containing 9 hub m7G-related lncRNAs (KRTAP5-AS1, LINC02804, GMDS-DT, ZDHHC20-IT1, LINC02688, MSC-AS1, PVT1, PCAT7, and CASC19). The m7Gscore could divide PCa patients into subgroups with significant differences in BCR-free survival. ROC curves also indicated that the m7Gscore has good prediction accuracy for BCR-free survival. We confirmed the stability and universality of the m7Gscore with the help of the test set, which contained the remaining 204 PCa patients from TCGA dataset. We also proved that the m7Gscore was an independent BCR-free prognostic marker for PCa patients. Then we established a nomogram model integrating the m7Gscore and several clinicopathological features (pathological T, Gleason score, and PSA), and we confirmed its better prediction ability. Its stability has also been verified in the test set and the entire TCGA cohort.

In GSEA, the high-risk group showed highly expressed immune activation pathways. Then, we evaluated the TIME of m7Gscore-related groups in PCa. In the ESTIMATE analysis, the immune score elevated and the tumor purity decreased in the high-risk group, which indicated a worse cancer prognosis and a higher chance of leading to immune evasion phenotype (40, 41). It is well known that CD8+ T cells have an antitumor effect, and the prognosis of PCa patients with high infiltration of CD8+ T cells and M2 macrophages was significantly improved (42). However, the high-risk patients with higher CD8+ T cells and M2 macrophage infiltration levels did not show better BCR-free survival. We speculated that it was due to the immunosuppressive microenvironment, which can be induced by the upregulated immune checkpoint, which was more likely formed in the high-risk patients (41). We also found elevated infiltration levels of DCs and Treg in the high-risk patients. It has been confirmed that both Treg and DCs can promote tumor progression by forming an immunosuppressive microenvironment (43, 44).

We further investigated the difference in somatic mutation in the m7Gscore-defined group. SPOP and TP53 were the most frequently mutated genes in the low- and high-risk groups, respectively. SPOP can target AR degradation and inhibit tumors in PCa (45). Its mutation will upregulate the assembly of stress particles to enhance the survival rate of PCa cells and docetaxel resistance (46). PCa with TP53 mutation is more likely to develop into an invasive tumor (47). The public sequencing data supported that high TMB indicated shorter BCR-free survival of PCa patients. The m7Gscore was significantly positively related to TMB and several immune checkpoints expression (PDCD1, CD274, PDCD1LG2, TIGIT, IDO1, and CTLA4). Immunotherapy is more likely to respond effectively to PCa patients with a high level of TMB or immune checkpoints (48, 49). Then we assessed the sensitivity to chemotherapy and endocrine drugs in the m7Gscore-defined groups. The results evaluated that bicalutamide, docetaxel, and vinblastine had more benefits for high-risk patients. ATRA, cisplatin, etoposide, gemcitabine, and methotrexate were more beneficial for the low-risk patients. Therefore, the m7Gscore has the potential to guide clinicians to individualize the drug treatment strategy for PCa patients.

Based on the expression of m7Gscore-related lncRNAs, 408 samples were clustered into three distinct m7G-related patterns. C1 had a significantly better BCR-free survival, and the survival of C3 was the shortest. Among the GSEA results, DNA damage repair functions were higher in C3, while adaptive immune activation and carcinogenic-related pathways were concentrated in C2. C1 showed a low expressed level of immune-activated functions. The immune score of C2 was also higher than that of C1 and C3. In the analysis of TIME, C2 was more highly infiltrated with most TIICs, and several immune checkpoints were also highly expressed. Similarly, C2 had higher expression in most immune-related functions. Given the high level of immune activation and TIIC infiltration of C2, we considered it as the immune-inflamed phenotype, while C1 with immunosuppression represents the immune-desert phenotype. Due to the characteristic of high TIIC infiltration and carcinogenic activation, C3 was considered the immune-excluded phenotype. Studies suggest that immune immunotherapy is more likely to exert an antitumor effect in the immune-inflamed tumor but has little effect on the immune-desert tumor, and the clinical effect on the immune-excluded tumor is uncertain (50).

This study still needs further exploration and verification. First of all, our study was completed based on TCGA cohort and lacked an independent cohort with sufficient lncRNA-sequencing data for verification. As this is a retrospective study, errors were more likely to appear in the process of information collection. Therefore, collecting an independent dataset with a sufficient sample size for multicenter prospective research to verify the accuracy and universality of the m7Gscore will make our research more convincing. Furthermore, the molecular mechanisms of hub m7G-related lncRNAs regulating translation in PCa need further explored *in vivo* or *in vitro* experiments.

## Conclusion

In conclusion, the m7Gscore made the m7G biological functions in PCa patients individualized and quantified. We comprehensively evaluated the correlation between the m7Gscore and BCR-free survival, clinicopathological stages, TIME, and somatic mutation. The m7Gscore can be applied as an independent marker for the BCR-free survival of PCa patients and also provide effective guidance for clinicians to formulate chemotherapy, endocrine therapy, and immunotherapy strategies. Finally, the three m7G-related patterns clustered based on the hub lncRNAs revealed the different immunophenotypes of PCa patients.

## Publisher’s Note

All claims expressed in this article are solely those of the authors and do not necessarily represent those of their affiliated organizations, or those of the publisher, the editors, and the reviewers. Any product that may be evaluated in this article, or claim that may be made by its manufacturer, is not guaranteed or endorsed by the publisher.

## Data Availability Statement

The original contributions presented in the study are included in the article/**Supplementary Material**. Further inquiries can be directed to the corresponding authors.

## Author Contributions

XL and WS designed the work and provided guidance. SX and YD participated in the data collection, data processing, figure preparation, and manuscript writing. JM and TW contributed to the statistical analysis. JL and SW contributed to the manuscript draft writing. XS revised the manuscript critically. All authors provided critical advice for the final manuscript.

## Funding

This research was funded by the National Natural Science Foundation of China (Grant Number: 82072838), Tongji Outstanding Young Researcher Funding (Grant Number: 2020YQ13), and Huazhong University of Science and Technology (Grant Number: 2019kfyXKJC06).

## Conflict of Interest

The authors declare that the research was conducted in the absence of any commercial or financial relationships that could be construed as a potential conflict of interest.

## Publisher’s Note

All claims expressed in this article are solely those of the authors and do not necessarily represent those of their affiliated organizations, or those of the publisher, the editors and the reviewers. Any product that may be evaluated in this article, or claim that may be made by its manufacturer, is not guaranteed or endorsed by the publisher.
