# Supplementary figures and images for: Characterization of 7-Methylguanosine Identified Biochemical Recurrence and Tumor Immune Microenvironment in Prostate Cancer
Source: Front Oncol. 2022 May 23;12:900203. doi: 10.3389/fonc.2022.900203 (PMC9168541; doi:10.3389/fonc.2022.900203)

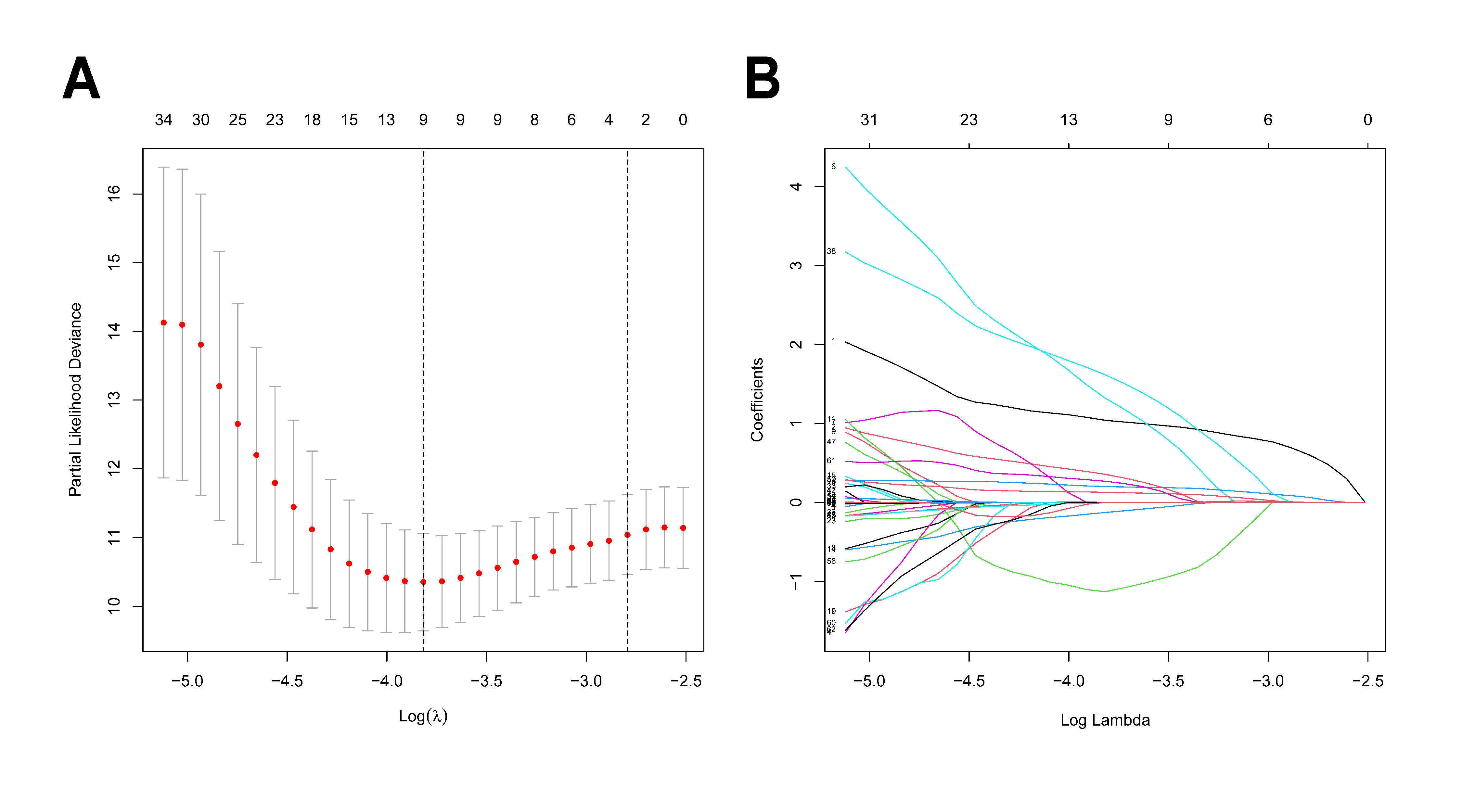

Supplement: Supplementary file 6 [file Image_1.tiff]

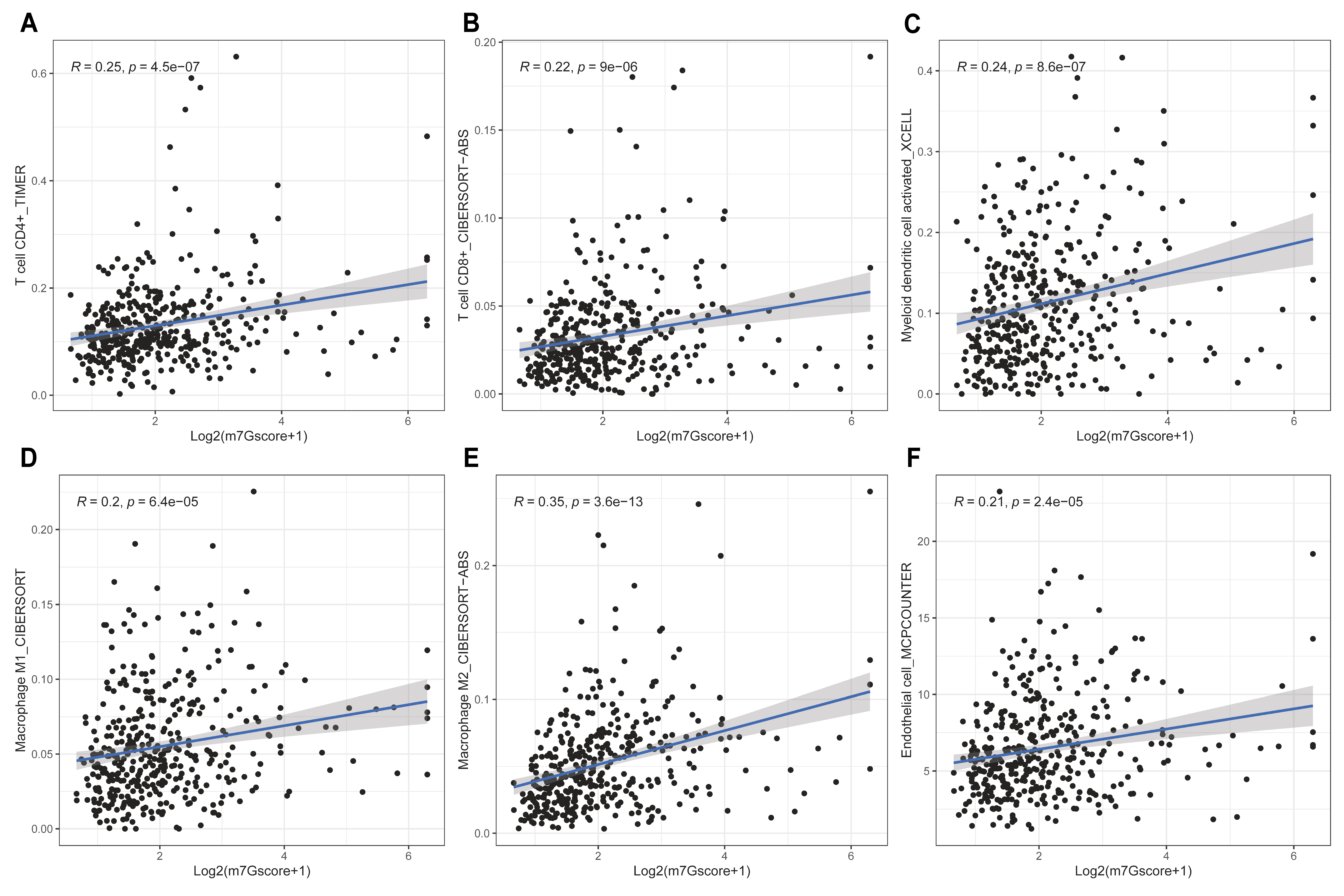

Supplement: Supplementary file 7 [file Image_2.tiff]
